# Multiplex Genome Editing in Chinese Hamster Ovary Cell Line Using All-in-One and HITI CRISPR Technology

**DOI:** 10.34172/apb.2021.032

**Published:** 2020-04-15

**Authors:** Fatemeh Safari, Safar Farajnia, Younes Ghasemi, Nosratollah Zarghami, Mazyar Barekati Mowahed

**Affiliations:** ^1^Medical Biotechnology Department, Faculty of Advanced Medical Sciences, Tabriz University of Medical Sciences, Tabriz, Iran.; ^2^Student Research Committee, Tabriz University of Medical Sciences, Tabriz, Iran.; ^3^Biotechnology Research Center, Tabriz University of Medical Sciences, Tabriz, Iran.; ^4^Department of Pharmaceutical Biotechnology, School of Pharmacy, and Pharmaceutical Sciences Research Centre, Shiraz University of Medical Sciences, Shiraz, Iran.; ^5^Department of Physiology & Biophysics, School of Medicine, Case Western Reserve University, Ohio, USA.

**Keywords:** Apoptosis, CHO cell, Caspase 7, CRISPR-Associated Protein 9

## Abstract

***Purpose:*** CRISPR/Cas9 gene editing technology has revolutionized gene manipulation by providing the opportunity of gene knock out/in, transcriptional modification and base editing. The application of this system extended into different eras of biology, from cell development to animal modeling. Various generations of CRISPR technology have been developed to make genome editing easy which resulted in rapid protocols for amelioration of a large genome.

***Methods:*** We established a simple protocol for gene manipulation in Chinese hamster ovary (CHO) cells to achieve a Caspase 7 deficient cell line by using combination of all-in-one CRISPR technology and CRISPR/Cas9 homology-independent targeted integration (CRISPR HITI).

***Results:*** the findings of this study indicated that using CRISPR knocking in/out technology facilitates genomic manipulation in CHO cells. Integration of EGFP in target locus of caspase 7 gene made the selection of knockout CHO cell line easy which achieved by cell sorting and single-cell cloning.

***Conclusion:*** this system introduces an effective targeting strategy for multiplex genome engineering, coinciding gene integration which simplified the selection of desired genomic characteristics.

## Introduction

Producing advantageous genomic characteristics in mammalian cell lines is among the highly precious strategies for gene-functional studies.^1,2^ Genome editing has been mostly carried out by using traditional methods such as RNA interference^3,4^ and homologous recombination. Nevertheless, the low frequency of desirable mutants and low specificity in addition to spontaneous cleavage of chromosomal DNA has resulted in the invention of site-specific nucleases. Recently, clusters of regularly interspaced short palindromic repeats (CRISPR)/CRISPR-associated (Cas) systems have opened a promising window for rapid and efficient gene editing at the specific genomic sites.^5,6^ The CRISPR/Cas9 system is composed of two components: guide RNA (gRNA) and Cas9 protein. Nuclease activity of Cas9 protein induces DNA double-strand breaks (DSBs) in any genomic region which is recognized by a gRNA. This gRNA must be accompanied by an adjacent protospacer motif (PAM) sequence in the target locus.^7,8^ NGG is a PAM sequence of Streptococcus pyogenes Cas9 (SpCas9) as the most versatile Cas9 protein.^9,10^

The CRISPR technology has been used for the manipulation of various mammalian cell lines, such as Chinese hamster ovary (CHO) cells. CHO cell line is a primary expression system that is widely utilized for the production of therapeutic pharmaceutics.^11^ These cells are the host for producing approximately one third of all biopharmaceuticals approved by the Food and Drug Administration (FDA) since 1982.^12^ Hence, the amelioration of this mammalian expression system is of great commercial interest. In order to respond to the demands of the market for large scale production of biologics, CHO cells must be grown in large bioreactors at high densities. This high density of cells leads to an environmental perturbation, and induces cell stress due to the limitation of nutrients and oxygen as well as the accumulation of toxic metabolic products. Sever and continuous stress prompts cell death by one of the two pathways, including passive cell death called necrosis and apoptosis as programmed cell death.^13^ Cell death by apoptosis is identified by specific morphological characters and activation of a variety of cellular signaling cascades. All death-inducing pathways need the involvement of specific downstream caspase effectors. Caspases are classified into two groups including initiator caspases (caspases 8, 9, 10 and 12) and executor caspases (caspases 3, 6 and 7). Active caspase 3 and 7 break a large set of substrates, which result in the characteristic morphological and biochemical clues of apoptosis including release of phosphatidylserine, nuclear condensation and genomic DNA fragmentation.^14^ Findings suggested that caspase-7 is responsible for cell detachment and ROS production.^15^ Hence, alteration of this gene may interfere with downstream pathways of apoptosis resulting in an apoptosis-resistant cell line. The application of this genome engineered cell line for the expression of therapeutic proteins may enhance the yield and decrease the final cost of biopharmaceutical products.

In the past decade, various engineered CHO cell lines have been established by using CRISPR/Cas9 technology.^16^ Cas9 nuclease can target multiple-locus due to the coincidental recognition of multiple PAM sequences^11^. This feature provides an opportunity for multiple recognition of targets by using multiple gRNAs beside a Cas9 protein.^17^ Since the co-transfection of numerous plasmids can lead to insufficient transfection efficiency of targeted cells, Sakuma and his colleagues developed a smooth and effective construction system called “all-in-one CRISPR/Cas9 systems”. This system, which is composed of vectors, expresses the Cas9 protein as well as up to seven gRNAs that are in tandem ligated into a single vector employing the Golden Gate assembly method.^18^

Gene silencing mediated by the CRISPR system is achieved via different strategies such as induction of point mutation, CRISPR excision, CRISPR homology-directed repair (CRISPR-HDR), and CRISPR/Cas9 homology-independent targeted integration (CRISPR HITI).

**Figure 1:**
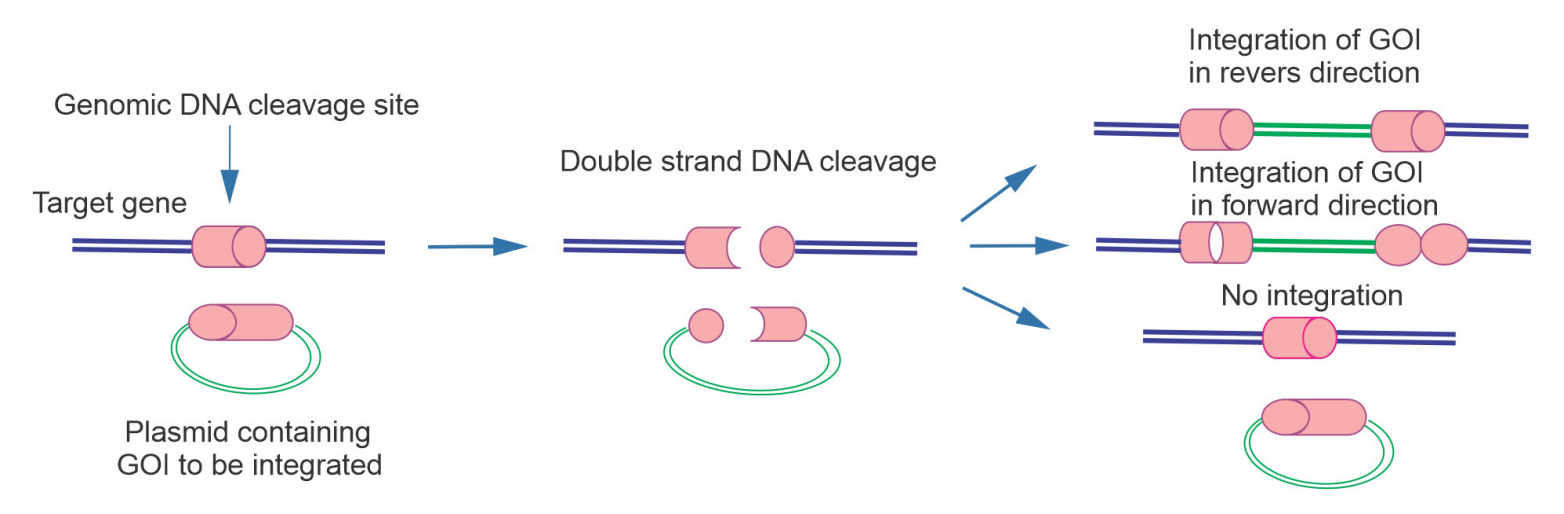
Scheme of HITI technology, which uses NHEJ-mediated targeted integration. In this method, Cas9 cleaves both genomic sequence and bait vector which harboring gRNA. The gRNA in bait vector is preceded by PAM sequence. This cleavage may repair in 3 conditions include 1: without gene integration 2: with integration in reverse direction (in these conditions DNA undergoes additional cleavage), 3: forward gene integration (gRNA can no longer bind to target cleavage sequence due to errors from NHEJ repair).

CRISPR excision can be defined as the complete elimination of a DNA segment surrounding an exon. In CRISPR HDR, the desired DNA construct is inserted in the genomic region site specifically by the HDR mechanism. CRISPR HITI employs an NHEJ repair pathway and CRISPR/Cas9 system to replace the genomic sequence located between two DSBs formed by Cas9 with the targeting fragment flanking on the bait vector (Figure 1).^19^ The invention of HITI facilitates knock out/in genetic modifications of primary and differentiated cells both in vitro and in vivo.^20^

In the following lines, we will be describing the protocol for using the combination of the CRISPR multiplex system and CRISPR HITI technology, which resulted in a caspase-7 knockout CHO cell line.

## Materials and Methods

Competent *E. coli* DH5a cells (Pasture Institute, Tehran, Iran) have been used for transformation of plasmids. Adherent CHO-K1 cell line was purchased from Pasture institute, Tehran, Iran.

Chemicals used in this study include Sodium chloride (Merck, USA), Deoxyribonucleic acid sodium salt (Merck, USA), Bacto yeast extract (Merck, USA), Bacto peptone (Thermo Fisher Scientific, USA), Bacto tryptone (Thermo Fisher Scientific, USA), sodium dodecyl sulphate (Merck, USA), hydrochloric acid (Merck, USA), EDTA (Merck, USA), 100% ethanol (Merck, USA), 100% glycerol (Merck, USA).

DNA purification kits (Dena Zist, Mashhad, Iran) and Gene All Expin^TM^ miniprep kit, (GeneAll, Korea) were the molecular biology reagents that used in this study.

All in one CRISPR system used in this study was containing pX330S-2 (Plasmid #58778, Addgene, USA), pX330A-1x2 (Plasmid #58766, Addgene, USA). We use PX460-1 containing U6 promoter-sgRNA insertion site-sgRNA scaffold and CAG promoter-enhanced GFP (EGFP)-bovine growth hormone polyadenylation signal (Dena Zist Company, Mashhad, Iran) as the HITI vector. The selection markers that use for colony selection were Ampicillin (Sigma-Aldrich, USA) and Spectinomycin hydrochloride (Sigma-Aldrich, USA). Primers used in this study are listed in Table 1.

**Table 1:** The sequences of gRNAs and primers

**Name of primers**	**Sequences**
Exon 4 gRNA	Fwd: CACCgAGATGGCGTGACGCCAATAA Rew: AAACTTATTGGCGTCACGCCATCTC
Exon 4 Bait gRNA	Fw: caccgCCTTTATTGGCGTCACGCCATCT Rev: aaacAGATGGCGTGACGCCAATAAAGGc
Exon 5 gRNA	Fwd: CACCGATACGCTTTAGGCATGCCG Rew: AAACCGGCATGCCTAAAGCGTATC
Exon 5 Bait gRNA	Fw: caccCCTCGGCATGCCTAAAGCGTATC Rev:aaacGATACGCTTTAGGCATGCCGAGG
CRISPR step2	Fwd: GCCTTTTGCTGGCCTTTTGCTC Rew: CGGGCCATTTACCGTAAGTTATGTAACG

Fast Digest BbsI (BpiI) (Thermo Scientific, USA, FD1014), Fast Digest Eco3I (Bsa1) (Thermo Scientific, USA, FD0294), T4 DNA Ligase (Life Technologies, USA, EL0014), BbsI (BpiI) (Thermo Scientific, USA, EL1012) are the restriction enzymes that have been used for gRNA cloning.

LB medium containing 5 g sodium chloride, 2.5 g Bacto yeast extract, 5 g Bacto tryptone, and up to 500 mL ddH_2_o was use for liquid bacterial culture. For making solid medium, 1% (w/v) Bacto agar was added and sterilized by heating for 20 minutes at 121°C.

DMEM (Gibco, USA) supplemented with 10% FBS was used for CHO cell culture. For transformation of CHO cells by CRISPR vectors, we applied Fermentas, USA).

### gRNA designing for multiplex targeting 

The specificity of Cas9 is identified by the gRNA sequence, which works as a guide for Cas9 to cleave the target sequence. In the case of SpCas9, the target sequence must immediately be preceded by a PAM sequence (5′NGG). The 20-nt guide sequence must be complimentary with the opposite strand to trigger Cas9 cleavage at ~3 bp upstream of the PAM. It is worth pointing out that the PAM sequence is needed to be present in the target DNA sequence, but not in the 20-nt guide in the gRNA.^21^ The specificity of gRNA is a critical issue; hence the selection of gRNAs with minimum off-target activity is necessary.^22^

Also, the gene targeting strategy is another significant issue. Various strategies have been used for gene targeting by CRISPR systems such as CRISPR HDR, CRISPR excision, and CRISPR HITI. Since the selection of knock out cells becomes a tedious task in CRISPR excision, we employed CRISPR HITI, which does not depend on the existence of homology arms and also makes single-cell selection easy. This technology allows the insertion of transgenes into both proliferating and non-proliferating cells efficiently because it is a cell cycle-independent strategy.^20^

To this end, the multiplex CRISPR system containing two gRNAs were constructed for targeting two genomic sites on the caspase-7 gene. Furthermore, gRNAs harboring genomic PAM was cloned in bait vectors. Nuclease activity of Cas9 resulted in the DNA cleavages in both genomic sites and bait vectors. Activation of the repair system subsequently integrates the cleaved sequence of bait vector (containing GFP) into a genomic site.

When it comes to the induction of an efficient mutation, the targeting locus is another critical issue. It is recommended to target CDS (coding DNA sequence) regions or the splicing sites of genes or active sites of protein.^23,24^ In this study, to augment the precision of gene disruption, the active site of caspase-7 flanking between exon 4 and exon 5 was targeted.

In order to select an appropriate gRNA with the minimum off-target activity, various on line soft wares with diverse sensitivity were used (Table 2). Desired loci were used as an input for these soft wares. Outputs with the highest on-target activity and the lowest unintended effects were selected. Because the U6 RNA polymerase III promoters (flanked in PX330 vector) prefers a guanine (G) nucleotide as the first base of its transcript, so in order to express the gRNA an extra G is added at the 5′ end where the guide sequence does not begin with G.^25^

**Table 2:** gRNA designing soft wares

**Web base tools**	**Address**
CRISPR Design Tool	https://crispr.mit.edu
Cas-OFFinder	http://www.rgenome.net/cas-offinder/
CRISPy	http://staff.biosustain.dtu.dk/laeb/crispy/
ChopChop	https://chopchop.cbu.uib.no/
CRISPR direct	https://crispr.dbcls.jp/
CCTop	https://crispr.cos.uni-heidelberg.de/

### Plasmid construction and cloning of gRNAs

#### Annealing the oligonucleotides 

To generate the CRISPR/Cas9 plasmids against caspase-7, top and bottom oligonucleotides were synthesized in a final concentration of 100 μM and phosphorylated and annealed in the mixture containing gRNA top and bottom (1 μL), T4 ligation buffer (1 μL), T4 PNK 1 μL and ddH2O (6 μL).

The reaction tube containing the mixture was put in the thermocycler with the following characteristics: 37°C (30 minutes); 95°C (5 minutes); ramp down to 25°C at 5°C min^–1^. The dilution of phosphorylated and annealed gRNAs was carried out by adding 1 μL of oligo to 199 μL of deionized water.

### Cloning of gRNAs in all in one multiplex vector

The annealed oligonucleotides, pX330A 1-2/S-2 vectors, BpiI enzyme, Quick ligase, and T4 DNA ligase buffer were mixed in two steps including digestion and ligation for cloning of gRNAs in the target vectors. The digestion reaction mixture was containing pX330A 1-2/S-2 (8.5 μL), BpiI (0.5 μL) and 10x buffer (1 μL). This mixture was prepared and maintained at 37°C for 10 minutes. The ligation reaction was performed by using digested vector (10 μL), 10 µM annealed gRNA (5 μL), T4 DNA ligase buf (2 μL), Quick ligase (1 μL) and ddH2O (2 μL).

The mentioned mixture was transferred to a thermal cycler with reactions including 3 cycles of 37°C (5 minutes) and 16°C (10 minutes). Following the cycling reaction, extra BpiI digestion was carried out by adding BpiI (0.5 μL) and ddH2O (9.5 μL) to the previous tube and maintaining at 37°C for 1 hour. A list of the constructed plasmids with their contents is represented in Table 3.

**Table 3:** constructed vectors containing related gRNAs

**Vectors**	**Targeted locus**	**gRNA sequence**
pX330 S-2	Exon 4	Fwd: CACCgAGATGGCGTGACGCCAATAA
		Rew: AAACTTATTGGCGTCACGCCATCTC
pX330A1-2	Exon 5	Fwd: CACCGATACGCTTTAGGCATGCCG
		Rew: AAACCGGCATGCCTAAAGCGTATC

### Golden Gate cloning 

**Figure 2:**
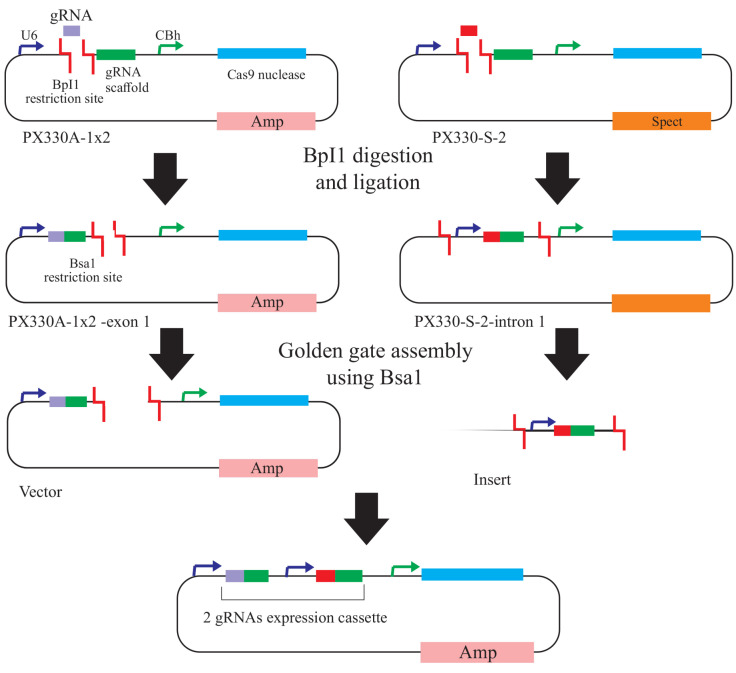
Schematic overview of the all-in-one CRISPR/Cas9 vector construction system for multiplex targeting of genome. STEP 1: Oligonucleotides relating to each target sequence are annealed and inserted into pX330A1-2 or pX330S-2 vectors by BpiI digestion. STEP 2: The constructed vectors bearing single gRNA expression cassettes are then assembled into an all-in-one vector containing multiple gRNA cassettes using the Golden Gate assembly method. Abbreviations: Amp, ampicillin; Spec, spectinomycin; U6, human U6 promoter; CBh, chicken beta-actin short promoter.

Golden Gate cloning was carried out by using pX330S-2 and pX330A1x2 plasmids 3μL, BsaI-HF enzyme (1 μL), Quick ligase (1 μL), T4 DNA ligase buffer (2 μL) and ddH2O (11 μL) in a single tube. This mixture was harbored a thermal cycling reaction including 4 cycle 37°C (15 minutes) and 16°C (30 minutes). Subsequent to the cycling reaction, extra BsaI-HF digestion was carried out at 37°C for 10 minutes (Figure 2).

The confirmation of plasmid construction was carried out by colony polymerase chain reaction (PCR) utilizing step2-F and step2-R primers (Table 1), followed by agarose gel electrophoresis.

### Cloning of bait gRNAs in CRISPR HITI vectors

The PX460-1 vector (2 μL), BpiI enzyme (1 μL), Gbuffer (1 μL) and ddH2O (16 μL) were mixed and maintained in 37°C over night to digest the vector and then used for ligation as follow. The ligation mixture was contained Digested pX460-1 vector (0.5 μL), 10 µM annealed gRNA (0.5 μL), 10×T4 DNA ligase buf (1 μL), Ligase buf (2 μL), ddH2O (16 μL). The mentioned mixture was maintained at 22°C overnight to ligate the bait gRNAs with the pX460-1 vector.

After each cloning, resulted plasmids were transformed into the competent DH5a strain, which followed by colony PCR to identified desired plasmids.

### Cell culture and transfections

CHOK1 cells were grown in DMEM medium supplemented with 10% FBS and antibiotics. Cells were incubated in a humidified incubator at 37°C and 5% CO2. One day before the transfection, cells were seeded in 6 well plates at 5-6 × 10^5^ density in antibiotic-free media. Cells were transfected with all in one vector encoding Cas9 and 2 gRNAs targeting the exons 4 and 5 of caspase-7 and bait vectors containing bait gRNAs and EGFP expression cassette. Each well was transfected with 3 μg DNA, respectively, using Lipofectamine 2000 with OptiMEM medium as transfection reagent based on the manufacturer’s recommendations. PmaxGFP® vector (Lonza, Basel, Switzerland) was used as control transfection reagent for confirmation of transfection efficiencies.

### Selection and detection of knockout alleles

A week after transfection, cell sorting was carried out by using FACS BD instrument. GFP expressing cells were seeded in 96 well plate by serial dilution to achieve a single cell clone. Following several passages, genomic DNA isolation and analysis was carried out for both knockout and wild-type cell lines followed by PCR amplification. Forward strand of exon 4 gRNA was used as a forward primer, and step2 R primer and the reverse strand of exon 5 gRNA were used as a reverse primers. PCR- products after clean-up and recovery reactions, were subjected to Sanger sequencing.

## Results

### Plasmid construction and cloning of sgRNA

**Figure 3:**
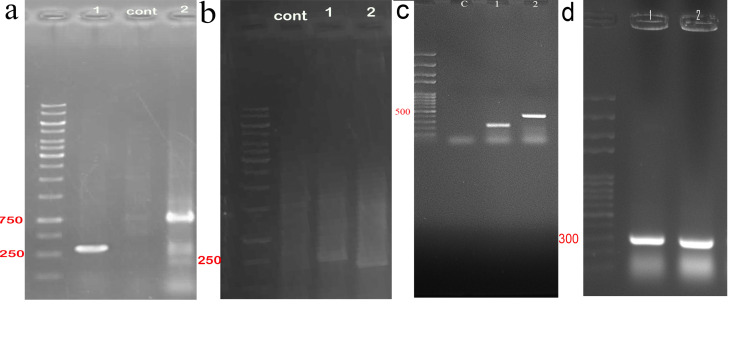
Gel electrophoresis of PCR product related to sgRNAs cloning in all in one and bait vectors. **(a)** exon 5 gRNA was cloned in PX330A 1x2, PCR products with the length of 229bp (lane 1 by using: step2 forward primer and reverse strand of exon 5 gRNA) and 680 bp (lane 3 by using: forward strand of exon 5 gRNA and step 2 reverse primer) confirmed this insertion. **(b)** exon 4 gRNA was cloned in PX330-s2, this insertion was validated by PCR product with the length of 229 bp (lane 1 by using: step2 forward primer and reverse strand of exon 5 gRNA) and 311bp (lane 2: by using forward strand of exon 4 gRNA and step 2 reverse primer). **(c)** construction of all-in-one vector was confirmed with the observation of 229 bp (lane 1 by using: step2 forward primer and reverse strand of exon 5 gRNA) and 458 bp (lane 1 by using: step2 forward primer and reverse strand of exon 4 gRNA) bands showed the presentation of both gRNAs in this vector. **(d)** this image refers to construction of bait vectors which contains each bait gRNA with the PCR product of 301 bp (lane 1 ,2 by using each forward strand of bait gRNAs and step 2 reverse primer).

Agarose gel electrophoresis followed by sequencing was applied to confirm the cloning of gRNAs within all in one and bait vectors. The cloning of exon 5 gRNA in Px330-A1-2 resulted in a PCR product with a length of 290 and 680 bp (Figure 3a). The cloning of exon 4 gRNA in PX330-s2 produced products with a length of 221 and 311 bp (Figure 3b). The construction of all in one vector confirmed by observing the PCR products with a length of 458bp and 226bp (Figure 3c). The PCR product validated the cloning of bait gRNAs in HITI vectors with a length of 301 bp (Figure 3d).

### CHO-K1 transfection, clonal selection and knockout clone detection

**Figure 4:**
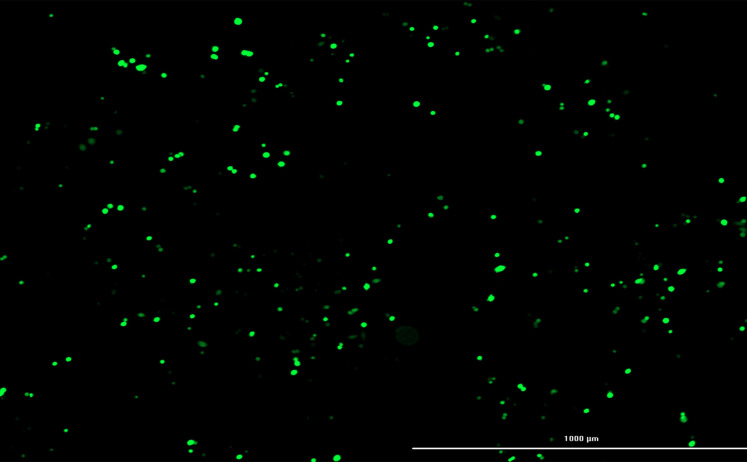
Transfection efficiency. Cytation^TM^ 5 Cell Imaging showed approximately 70% transfection efficiency.

Transfection efficiency of CRISPR plasmids was ascertained by Cytation^TM^ 5 Cell Imaging. The results of this imaging demonstrated that 70% of cells had been transfected successfully (Figure 4).

**Figure 5:**
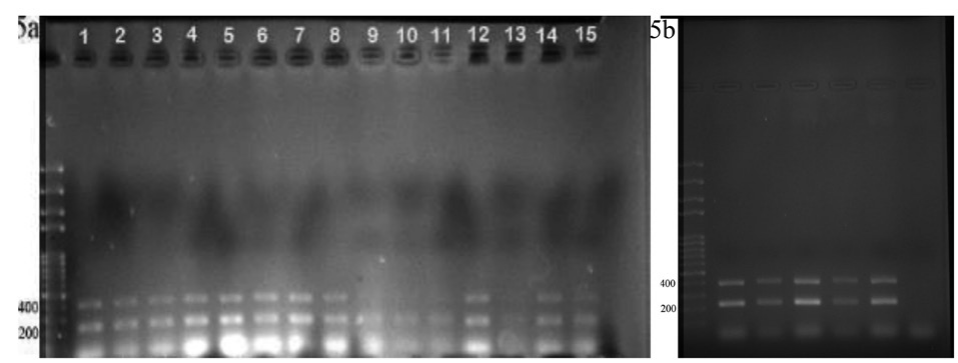
The confirmation of heterozygote knocked out allele. **(a)** This gel electrophoresis showed heterozygote clones contain one allele of wild type cells with the length of 395 bp and knock out allele with 220 bp length. (**b**) the results of second round of transfection represent the both 395 and 220 bp PCR product which confirmed the results of first round transfection.

**Figure 6:**
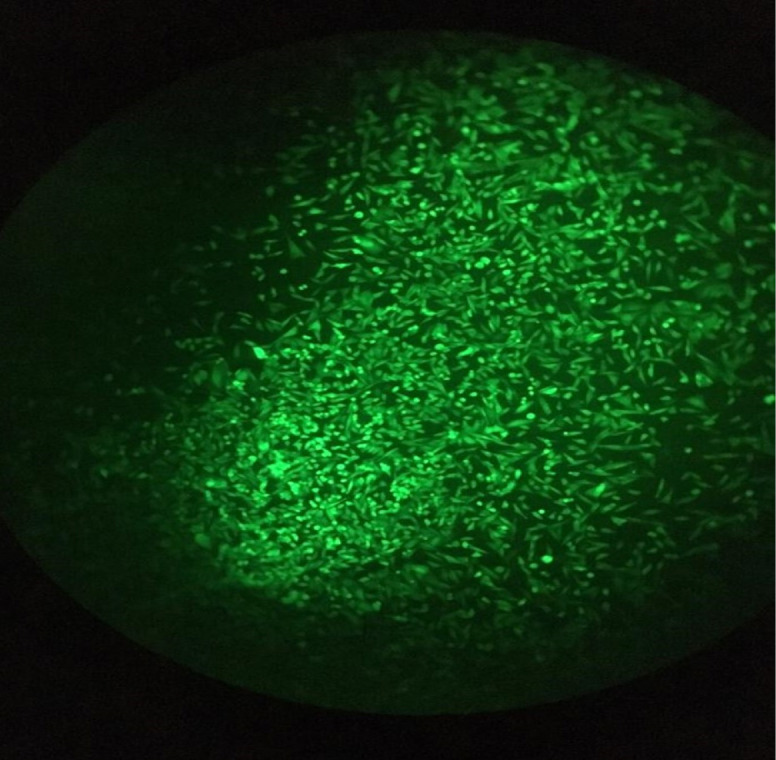
Heterozygote caspase 7 knock out CHO cell line expressing EGFP.

**Figure 7:**
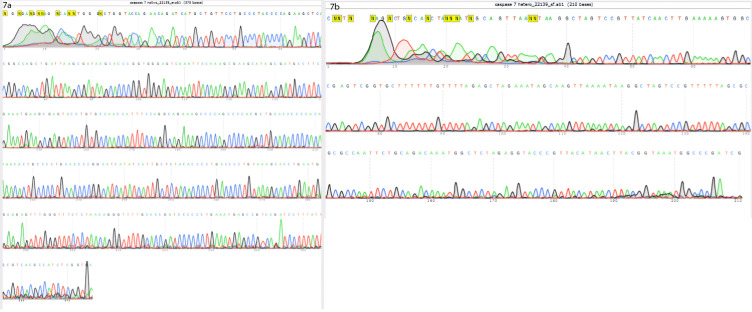
Findings of sequencing are represented in these figures: **(a)** the results of 395 bp PCR product sequencing showed the intact allele of CHO cell. **(b)** the results of 220 bp PCR product sequencing showed the integration of bait vector in one allele of CHO cell genome

Cell sorting (by using FACSCALIBUR, BD, USA) followed by single-cell cloning via serial dilution was used for clonal selection. Detection of knockout cells which expressed GFP was carried out by genomic DNA isolation and PCR for each single clone. EGFP integration was confirmed by observing a 220 bp band on gel electrophoresis, which belonged to the bait vector. Following the first round of transfection, results of PCR and gel electrophoresis showed that the selected clones were heterozygote by displaying both 395 bp band (which belonged to native genomic allele) and 220 bp (which belonged to knockout allele) PCR product (Figure 5a). From these clones, EGFP expressing clone (Figure 6) was selected and harbored the second round of transfection. The results of genomic PCR following the second transfection were similar to the first transfection and resulted in heterozygote caspase-7 knockout clone (Figure 5b). The findings of PCR product sequencing also confirmed the heterozygosity of clones (Figure 7a and 7b).

## Discussion

Genome editing using the CRISPR/Cas9 system is an efficient strategy for the establishing of genome-engineered cells. Multiplex genome editing is one of the most appealing approaches in gene modification today. This approach provides us with a simple and effective strategy for single vector that mediated multiple targeting.^26^ In order to produce knockout cells, various strategies have been at our disposal, including the CRISPR excision, CRISPR HITI and CRISPR HDR. For the execution of CRISPR excision, no donor vectors were used, and therefore no reporter genes were observed to be integrated into the genome. Hence, the selection of knockout cells becomes a repetitive task. In contrast, CRISPR HDR is executed by recruiting donor vectors thus allow the selection of the targeted cells based on resistance to antibiotic or observing fluorescence. It is noteworthy that this strategy requires the homology arms, which must be cloned into the bait vector. The CRISPR HITI does not depend on the existence of homology arms and facilitates the selection of the knockout cells. HITI approach empowers us to the direct invention of knockout cells; thus each cell with fluorescence phenotype is easily selected as an allele knockout.^19,27^

In comparison with the HDR, HITI is more eventful as it relies on the cell cycle phase independent mechanism of double-stranded DNA break repair (NHEJ). HITI provides a large percentage of cells with the desired DNA insertion in the correct orientation and a correct locus. However, certain limitations are recognized in HITI. Due to the design procedure, inevitably, additional nucleotides are introduced into the target locus accompanied by the insertion of the DNA template at the ends of the insert. Hence, special consideration should be paid to possible reading frame shifts as well as deletion of native or emergence of new regulatory sequences in the noncoding genomic locus.^28^

It is widely believed that caspase-7 is an executioner caspase with a role in the earlier stages of the apoptotic pathway. Findings have shown that the deletion of caspase-7 in different cell lines resulted in different cellular micro-environments. For example, disruption of caspase-7 is not lethal in chicken B cell lymphoma line DT40 and mouse embryonic fibroblasts cells.^29,30^ Nevertheless, the caspase-7 gene deletion is reportedly lethal in mice.^31,32^ On the same token, we found that homozygote caspase-7 deletion seemed to be lethal in the CHO cell line, which begets more study. These results raise the possibility that the effect of caspase-7 deficiency varies and cell line dependent.

## Conclusion

In conclusion, we delineated here the CRISPR/Cas9 mediated strategy that provides caspase-7 knockout CHO cell line. All in one CRISPR/Cas9 vectors facilitate the one-step generation of the mutant organism with multiple engineered genes. Furthermore, the HITI strategy is easy to use and can be employed for the production of knockout cells for a specific gene of interest. CHO cells are the popular mammalian workhorse for the generation of commercial therapeutically essential proteins. The development of recombinant CHO has become a priority in biopharmaceutical manufacture. Although, the resulted CHO caspase-7 knockout cell line in this study was a heterozygote, efforts are ongoing to attain a probable homozygote knockout cell. This can be materialized by using another selection marker such as puromycin, which is integrated into the target site by HITI strategy.

## Ethical Issues

Ethics approval for the study was obtained from an ethics committee of Tabriz University of Medical Sciences dated 2016/13/08, No: 5/4/46151.

## Conflict of Interest

The authors declare that they have no conflict of interest.

## Acknowledgments

Authors would like to thank the Department of Medical Biotechnology, Faculty of Advanced Medical Sciences, Tabriz University of Medical Sciences for supporting this project (grant No. 94/4-2/4). In addition, this study was financially supported by grant No: 950609 of the Biotechnology Development Council of the Islamic Republic of Iran.
